# The first complete genomic structure of *Butyrivibrio fibrisolvens* and its chromid

**DOI:** 10.1099/mgen.0.000216

**Published:** 2018-09-14

**Authors:** Javier Rodríguez Hernáez, Maria Esperanza Cerón Cucchi, Silvio Cravero, Maria Carolina Martinez, Sergio Gonzalez, Andrea Puebla, Joaquin Dopazo, Marisa Farber, Norma Paniego, Máximo Rivarola

**Affiliations:** ^1^​ Biotechnology Institute, CICVyA-Instituto Nacional de Tecnología Agropecuaria (INTA), Hurlingham, Provincia de Buenos Aires, Argentina; ^2^​ Fundación Universidad Argentina de la Empresa (UADE), Buenos Aires, Argentina; ^3^​ Skoklab - Department of Pathology, NYU Langone Health, New York, USA; ^4^​ Clinical Bioinformatics Research Area, Fundación Progreso y Salud, Hospital Virgen del Rocío, Sevilla, Spain; ^5^​ CONICET, Buenos Aires, Argentina

**Keywords:** *Butyrivibrio fibrisolvens*, cow rumen, genome sequencing, INBov1

## Abstract

*Butyrivibrio fibrisolvens* forms part of the gastrointestinal microbiome of ruminants and other mammals, including humans. Indeed, it is one of the most common bacteria found in the rumen and plays an important role in ruminal fermentation of polysaccharides, yet, to date, there is no closed reference genome published for this species in any ruminant animal. We successfully assembled the nearly complete genome sequence of *B. fibrisolvens* strain INBov1 isolated from cow rumen using Illumina paired-end reads, 454 Roche single-end and mate pair sequencing technology. Additionally, we constructed an optical restriction map of this strain to aid in scaffold ordering and positioning, and completed the first genomic structure of this species. Moreover, we identified and assembled the first chromid of this species (pINBov266). The INBov1 genome encodes a large set of genes involved in the cellulolytic process but lacks key genes. This seems to indicate that *B. fibrisolvens* plays an important role in ruminal cellulolytic processes, but does not have autonomous cellulolytic capacity. When searching for genes involved in the biohydrogenation of unsaturated fatty acids, no linoleate isomerase gene was found in this strain. INBov1 does encode oleate hydratase genes known to participate in the hydrogenation of oleic acids. Furthermore, INBov1 contains an enolase gene, which has been recently determined to participate in the synthesis of conjugated linoleic acids. This work confirms the presence of a novel chromid in *B. fibrisolvens* and provides a new potential reference genome sequence for this species, providing new insight into its role in biohydrogenation and carbohydrate degradation.

## Data Summary

This Whole Genome Shotgun project has been deposited at DDBJ/ENA/GenBank under accession GCA_003175155.1. All the sequencing data used in this experiment and assembly details are under NCBI BioProject PRJNA412083. https://www.ncbi.nlm.nih.gov/bioproject/PRJNA412083/

**Impact Statement**

Currently, all assembly projects available for *Butyrivibrio fibrisolvens* species provide the genome sequence information in more than 60 unordered sequences. In the present study, we assembled the complete genomic structure of *B. fibrisolvens* in one sequence. We identified ~96 % of the bases, and established the number and position of the ~4 % unidentified bases. Furthermore, we identified a chromid, the first sequenced chromid in this bacterial species. The results presented here will contribute to our understanding of the role of the *B. fibrisolvens* as part of the rumen microbiota.

## Introduction

*Butyrivibrio fibrisolvens* is part of the gastrointestinal microbiome of ruminants and other mammals, including humans. This species belongs to the genus *Butyrivibr*io (class *Clostridia*) which comprises non-spore-forming, monotrichous, anaerobic, butyric-acid-producing, curved rod-shaped bacteria [1]. It is ubiquitously present in the gastrointestinal tract of many animals and in high abundance in the bovine rumen, which suggests that this organism plays an important role in the ruminal fermentation of polysaccharides involved in cellulose degradation [2]. Therefore, *B. fibrisolvens* can provide genetic resources of potential use in the utilization of vegetal biomass for the development of third-generation biofuels. Moreover, this species participates in the biohydrogenation of polyunsaturated fatty acids in ruminants and has been proposed to improve the fatty acid profile of milk and meat from ruminant animals and thus the creation of healthier food products [3]. This species has also been evaluated in mice as a probiotic that prevents enterocolitis [4] and colorectal cancer [5].

As of now, there is still no closed circular genome sequence for *B. fibrisolvens* in any public databases (see Genome Properties section). There are nine genome assembly projects of *B. fibrisolvens* deposited in the NCBI genome database, each one in more than 60 unordered sequences. These genome sequences have been provided by the Hungate1000 Consortium [6] and the DOE Joint Genome Institute (JGI). One previous study [7], using DNA isolation, suggests that the *B. fibrisolvens* genome contains a chromid with an estimated molecular weight of 200 MDa. Nevertheless, this has not been confirmed by sequencing and the structure and function of this putative chromid remain unknown. Thus, the aim of this work is two-fold: to contribute to the production of a nearly complete genome sequence for the *B. fibrisolvens* INBov1 strain obtained from cow rumen, and to provide the first chromid sequence of this species, contributing new insight into its characteristics.

## Methods

### Genome sequencing and assembly

This genome assembly project was performed at Instituto Nacional de Tecnología Agropecuaria (INTA) within the bioinformatics unit. The results of this Whole Genome Shotgun project have been deposited at DDBJ/ENA/GenBank under accession GCA_003175155.1. Details of bacterial isolation, growth conditions and species characterization methods and results are available in File S1 (available in the online version of this article).

For genome assembly we obtained a complex dataset of reads from different instruments and from different library preparations, as well as an optical map restriction digest. Overall, we had ~750 000 sequences of Illumina paired-end (PE) reads (2×250 bp) produced by the sequencing services using Miseq (Illumina) performed at INTA, Instituto de Biotecnología (Argentina), Consorcio Argentino de Tecnología Genómica (CATG). Additionally, we had 220 000 single-end (SE) reads (300 bp in length) and 200 000 mate pair (MP) reads (300 bp in length, insert size ~2000 bp) which were obtained through GS 454 FLX technology (Instituto Indear of Rosario, Argentina). In addition, we created an optical restriction map of *B. fibrisolvens* INBov1 with the enzyme *Kpn*I to aid in scaffold ordering (OpGen Technologies). Quality control was tested using FastQC [8] and the Illumina PE and 454 SE reads were trimmed using Trimmomatic [9]. Almost half of the Illumina PE reads were extended using the FLASh program [10].

A detailed description and discussion of the assembly methods and genome annotation is included in File S1.

## Results

### Assembly and genome organization

**Fig. 1.**
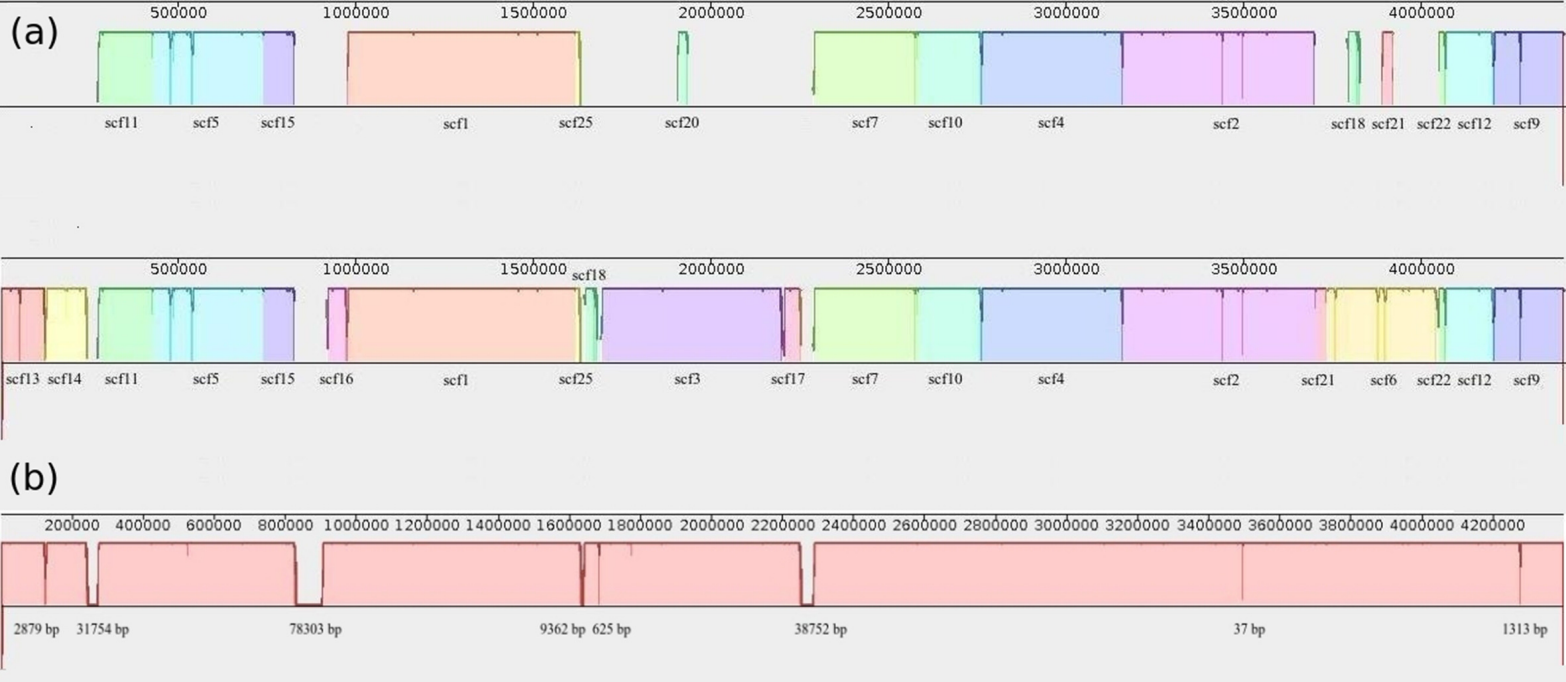
(a) Top: visualization using the program Mauve [18] of scaffolds placed by Soma in the optical restriction map (70 % map coverage). Bottom: structure of the genome sequence after manual placing of scaffolds with NEBcutter (95 % map coverage). (b) Final genomic sequence of *B. fibrisolvens* (4 398 850 bp, complete genomic structure with 96 % of identified bases) after using GapCloser. Gap length is shown (gap regions smaller than 10 bases are not shown).

After analysis of the different assembly trials tested (see ‘Assembly discussion’ in File S1), the final workflow chosen to reconstruct the genome was via the Newbler software because fewer scaffolds were produced and higher map coverage values were obtained through this workflow. Map coverage refers to the percentage of sequence assembled which aligns in accordance with the restriction map provided by the optical mapping results. The 25 scaffolds obtained with Newbler with only 454 reads (SE and MP) were used to reconstruct the genome sequence. Soma, a scaffold-restriction map aligner pipeline, placed 15 scaffolds in the optical map alignment. In a following step, after further analysis using NEBcutter [11], six new scaffolds were placed by manual alignment. We also relocated two small scaffolds and removed one (see Fig. 1a). A complete description of the manual alignment process is described in the Supplementary Material.

In order to reduce the number of gaps in the scaffolds, we performed gap filling with the Illumina PE reads. GapCloser [12] was used with the Illumina PE reads to close 93 % of the gaps present in all of the scaffolds (41 570 total bases). Moreover, the genome size was estimated from the optical map restriction data, which gave a value of 4 327 514 bp. This was similar to the size estimated from the *kmer* distribution, using the Illumina PE reads, which was 4 407 001 bp (see File S1). Overall, we assembled close to 96 % of the genome sequence in one scaffold of 4 398 850 bp and had only 163 074 unidentified bases (see Fig. 1b). The position and number of the unidentified bases was established by alignment of the MP reads and by positioning the scaffolds in the restriction map. Because the position and number of the unidentified bases could be determined, we were able to complete the full genomic structure of the genome. We also identified one large unplaced scaffold (266 542 bp) as a new putative chromid. This scaffold did not align with any region of the restriction map and, among other plasmidic features, contained a *repA* gene, which encodes a plasmid replication initiator protein [13, 14] (see ‘Genome insights from the genome sequence’ section).

### Genome properties and statistics

The genome of strain INBov1 contains one scaffold of 4 279 765 bp (163 097 gaps), representing 96 % of the estimated complete genome sequence and the complete genomic structure. One chromid in one sequence of 266 542 bp (pINBov266), four scaffolds in a range of ~2–107 kbp (total of 174 701 bp) and 64 small contigs smaller than ~2 kbp (total of 41 201 bp) remained unplaced but all contained at least one annotated gene. The total size of the non-redundant genome data set is 4 721 197 bp, including the chromid sequence (4 457 655 bp without counting the chromid). The RAST server (http://rast.nmpdr.org) [15] annotated, including all the sequences, 4027 coding sequences that correspond to 3947 proteins and 80 RNAs. The G+C content calculated by RAST is 39.9 %. According to COG annotation, 3121 genes (including 155 genes encoded in the chromid) were classified by using the WebMGA server [16] (see Table 1).

**Table 1.** COG annotation statistics

COG code	Genes	Percentage of total genes	Description
J	174	4.4	Translation, ribosomal structure and biogenesis
A	0	0.0	RNA processing and modification
K	214	5.4	Transcription
L	164	4.2	Replication, recombination and repair
B	0	0.0	Chromatin structure and dynamics
D	52	1.3	Cell cycle control, cell division, chromosome partitioning
V	103	2.6	Defence mechanisms
T	186	4.7	Signal transduction mechanisms
M	223	5.7	Cell wall/membrane biogenesis
N	32	0.8	Cell motility
U	30	0.8	Intracellular trafficking and secretion
O	78	2.0	Post-translational modification, protein turnover, chaperones
C	110	2.8	Energy production and conversion
G	322	8.2	Carbohydrate transport and metabolism
E	172	4.3	Amino acid transport and metabolism
F	84	2.1	Nucleotide transport and metabolism
H	107	2.7	Coenzyme transport and metabolism
I	63	1.6	Lipid transport and metabolism
P	110	2.8	Inorganic ion transport and metabolism
Q	11	0.3	Secondary metabolite biosynthesis, transport and catabolism
R	372	9.4	General function prediction only
S	249	6.3	Function unknown
–	266	6.7	Multiple classes
–	1092	20.9	Not in COGs

The statistics obtained with the genome of strain INBov1 are very similar to those observed in the other *B. fibrisolvens* genomes deposited in the NCBI genome database. Median values for these genomes are 4.7 Mb for genome size, 39.7 % for G+C content and 3764 for proteins annotated. The exception is the genome of strain 16/4 (GenBank: GCA_000209815.1), the metrics of which differ considerably and this strain behaves as an outlier; it presents a genome size of 3.16 Mb, G+C content of 38.6 % and 2966 proteins annotated. Therefore, we also evaluated the 16S rRNA gene sequence of strain 16/4 (GenBank: AJ250365.2) to assess its species identity. The results obtained by using the identification service of the EzBioCloud database [17] showed that the closest species to strain 16/4 is *Pseudobutyrivibrio ruminis* DSM 9787^T^ (GenBank: X95893) with a sequence similarity of 98.18 %. The level of sequence similarity between the 16S rRNA genes of strain 16/4 and *B. fibrisolvens* NCDO2221^T^ (GenBank: X89970.1) is 88.56 %, considerably lower than the species threshold proposed by several authors [18, 19]. This suggests that strain 16/4 might have been incorrectly classified as a member of *B. fibrisolvens* by NCBI.

**Fig. 2.**
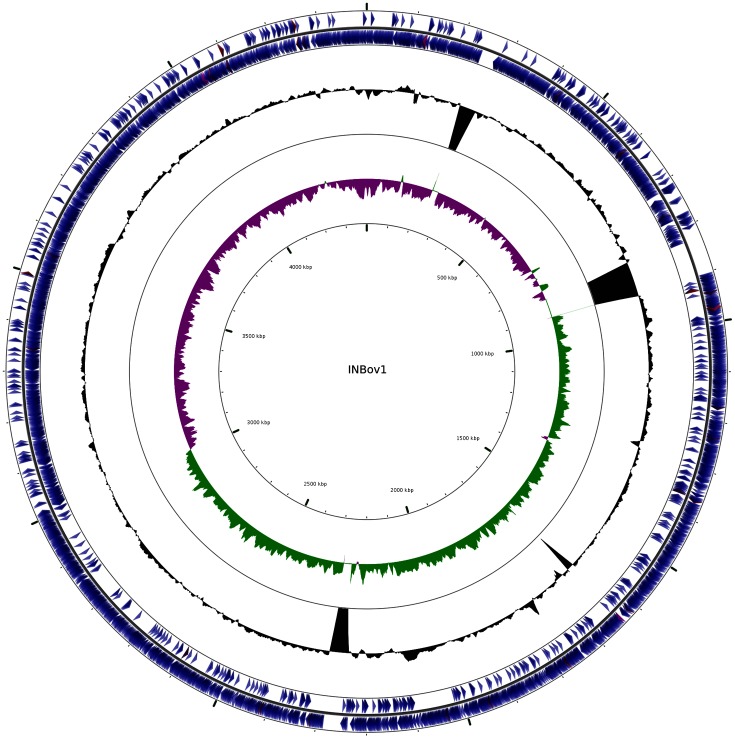
Circular visualization of the *B. fibrisolvens* INBov1 chromosome. The image shows (from outside to centre): genes on the forward strand, genes on the reverse strand (coding sequences in blue, tRNAs in red, rRNAs in purple). The G+C content is in black with peaks indicating higher or lower values than the average G+C content (peaks out/inside, respectively). There are four noticeable peaks inside that correspond to the largest gap regions, as shown in Fig. 1. The inner circle shows the GC skew. Positive values correspond to green peaks, indicating that the amounts of guanines are enriched in the top strand versus the amount of cytosines in the bottom strand. Purple peaks represent the opposite.

In Fig. 2 the genome sequence of INBov1 is visualized by using CGview [20]. The GC skew shows a characteristic asymmetry in the nucleotide frequency present in most prokaryotes where a higher frequency of guanines is found in the leading strand [21], in accordance with the *Theta* replication model. Therefore, the origin of replication and the site of termination of the genome are generally located in regions where the skew in the GC content shifts. This GC skew adds further confidence that this is a well-assembled genome.

### Insights from the genome sequence

Following annotation of the INBov1 genome, we focused on analysis of carbohydrate active enzyme (CAZymes) families due to their potential in many biotechnological applications. We also performed a comparative analysis of the genomes and carbohydrate enzymes of INBov1 and the other species of the genus *Butyrivibrio*: *Butyrivibrio hungatei* MB2003, *Butyrivibrio proteoclasticus* B316 and *Butyrivibrio crossotus* DSM 2876 (GenBank IDs: GCA_001858005.1, GCA_000145035.1 and GCA_000156015.1). We used the Carbohydrate Active Enzymes database (http://www.cazy.org) [22] and the DBCan server (http://csbl.bmb.uga.edu/dbCAN/) [23] to annotate the INBov1 enzymes.

INBov1, as expected, encodes an extensive repertoire of CAZymes with 114 glycosyl hydrolases (GHs), 33 carbohydrate esterases (CEs), three polysaccharide lyases (PLs) and 86 glycosyl transferases (GTs) encoded in the genome, indicating that INBov1 has a similar distribution of CAZymes to *B. proteoclasticus* B316. Moreover, INBov1 and *B. proteoclasticus* B316 are also similar in terms of genome size and number of genes.

INBov1 has a role in the biohydrogenation of unsaturated acids. However, a gene encoding linoleate isomerase (EC 5.2.1.5) was absent from this strain. Interestingly, INBov1 does encode an oleate hydratase (EC 4.2.1.53) involved in the biohydrogenation of oleic acids. No linoleate isomerase genes were found in the other *Butyrivibrio* species, and oleate hydratase genes were only found in *B. crossotus*.

The INBov1 genome encodes a full-length enolase (EC 4.2.1.11), which is present on the main chromosome. This glycolytic pathway enzyme, also known as phosphopyruvate hydratase, is responsible for the conversion of 2-phosphoglycerate (2 PG) to phosphoenolpyruvate (PEP). Enolases have recently been linked to the biohydrogenation of linoleic acid in *Lactobacillus plantarum* [24]. Genes encoding enolase were also present in *B. hungatei* and *B. crossotus.*

INBov1 encodes two l-lactate dehydrogenase (EC 1.1.1.27) genes, one on the main chromosome and the other on its chromid. A gene encoding this enzyme was also found in *B. hungatei*. Genes encoding enolase and l-lactate dehydrogenase are co-localized in the INBov1 genome, an observation that is consistent with a recent study [25] on the rumen microbiome of members of the Hungate1000 Collection.

INBov1 lacks genes encoding two key glucose metabolism enzymes, namely d-glucose phosphotransferase (EC 2.7.1.199), involved in glucose uptake, and phosphoglucomutase (EC 5.4.2.2), required for the inter-conversion of d-glucose 1-phosphate to d-glucose 6-phosphate. Genes encoding phosphoglucomutase were, however, present in *B. proteoclasticus* and *B. hungatei*. In contrast, INBov1 does have a phosphomannomutase (EC 5.4.2.8) and genes encoding this enzyme were found in all the *Butyrivibrio* species with the exception of *B. crossotus*. An interesting finding is that INBov1 and *B. proteoclasticus* encode copies of this gene on both their main chromosome and their chromids.

The ubiquitous presence and high abundance of *B. fibrisolvens* in ruminants suggest that this species plays a significant role in cellulose degradation. Consequently, we characterized the INBov1 genes involved in the cellulolytic process. The glycosyl hydrolases that play a major role in cellulolysis are the endoglucanases (EC 3.2.1.4), β-glucosidases (EC 3.2.1.21) and exoglucanases, which include celodextrinases (EC 3.2.1.91) and cellobiohydrolases (EC 3.2.1.176; EC 3.2.1.74) [26]. Other non-glycosyl hydrolase enzymes have also recently been found to participate in cellulolysis. Laccases (EC 1.10.3.2) and peroxidases (EC 1.11.1) have been shown to participate in the degradation of lignin. Polysaccharide monooxygenases (PMOs) and lytic polysaccharide monooxygenases (LPMOs) also play a role in the cellulose decrystallization process [26].

The INBov1 genome encodes 41 genes related to cellulolytic processes. Among these were several genes encoding endoglucanase and β-glucosidase. The endoglucanase genes were only found on the main chromosome, while genes encoding β-glucosidases were found on both the chromosome and the chromid; a similar situation was observed in *B. proteoclasticus*. Endoglucanase genes were found in all *Butyrivibrio* species, while β-glucosidase genes were found in all *Butyrivibrio* species except *B. crossotus*. Interestingly, exoglucanase genes were absent, not only from INBov1, but from all *Butyrivibrio* species. As expected, no lignin degradation genes, PMO or LPMO genes were found in any of the *Butyrivibrio* species.

### Chromid replicon

An important feature of the INBov1 genome was the presence of a single large unplaced contig that we identified initially as a mega-replicon (pINBov266). As a result of a detailed analysis of this contig and its gene content, we have reclassified this replicon as the first chromid to be identified in *B. fibrisolvens*. Chromids are defined as replicons with a G+C content that is similar to the main chromosome. However, they have plasmid-type maintenance and replication systems and are significantly smaller than the main chromosome [14].

**Fig. 3.**
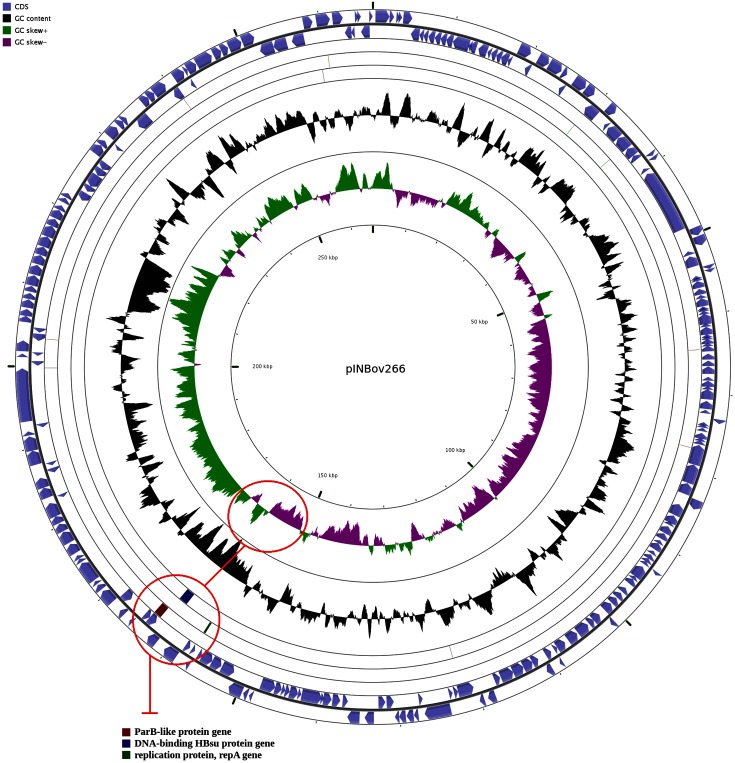
Circular visualization of the chromid sequence (pINBov266) in Cgview. The location of *parB*, *hbs* and *repA* genes are shown by red circles. The upper circle shows that these genes are located in a region where the GC skew switches the nucleotide frequency polarity. The lower circle corresponds to the blast hits of the genes *parB* (red), *hbs* (blue) and *repA* (green).

The G+C content of the chromid is 38.9 % and it contains 238 coding sequences, including the genes related to plasmid replication systems (e.g. *repA*, *parB* and *hbs*). RepA is a motor protein that acts as an initiator factor for plasmid replication [13, 14]. The *parB* gene encodes a centromere-binding protein (CBP), an element characteristic of type 1 partition systems [27]. The hbs protein was shown to participate in controlling DNA gyrase activity [28, 29], playing a role in the initiation of oriC-dependent DNA replication [30, 31]. The *repA*, *parB* and *hbs* genes are co-localized in a region where the GC skew switches the nucleotide frequency polarity (Fig. 3), suggesting that the origin of replication might be located in that area. No conjugation-related genes (e.g. *tra* and *trb* genes) were found in the chromid, main chromosome or any unplaced contig sequences. Moreover, use of the oriTfinder tool [32] failed to find evidence of an origin of transfer (oriT) in the pINBov266 sequence. As a result, we conclude there is no conjugative system in pINBov266, suggesting that it is likely to be non-mobile.

pINBov266 encodes several genes involved in antibiotic resistance, and the production of bacteriocins and toxins. We found genes encoding multi-antimicrobial extrusion protein genes from the MATE family of MDR efflux pumps, known to be crucial for resistance to antimicrobial compounds [33]. The chromid also encodes genes for β-lactamase, VanZ and ABC-transporters, which are also involved in antibiotic resistance, and for MerR and SpaF/MutF genes, which are involved in cobalt–zinc–cadmium and lantibiotic bacteriocin resistance, respectively [34, 35].

Chromid pINBov266 encodes 35 putative carbohydrate degradation enzymes, including 17 different types of hydrolases (including a serine hydrolase, α/β hydrolase and glycoside hydrolase). Genes encoding four key enzymes in the glycolysis/gluconeogenesis pathways were also identified: phosphotransferase (EC 2.7.1.90), phosphohexokinase (EC 2.7.1.11), l-lactate dehydrogenase (EC 1.1.1.27), aldehyde dehydrogenase [NAD(P)+] (EC 1.2.1.5), and the only aldehyde dehydrogenase (NAD) (EC 1.2.1.3) gene present in the entire genome. An interesting finding was the presence of genes on the INBov1 genome encoding two PLs of which the PL9 and PL11 genes are encoded only in the chromid. SignalP [36] analysis indicates that the products of these PL genes appear to be secreted.

Genes encoding proteins of the NiFe hydrogenase maturation system (HypD, HypE, HypF and Ferredoxin subunit A) are also only present in pINBov266. Four of the five total genes, which play a role in the hydrogenase maturation system, are found only on the chromid. This system is known to provide a mechanism to store and utilize energy by reversibly converting molecular hydrogen, one of the key products of rumen fermentation [37, 38]. Furthermore, pINBov266 encodes proteins from the TldE/TldD proteolytic complex, which have been reported to play a key role in the maturation and exportation of antibiotics and other proteins [39]. Other genes that were unique to pINBov266 included genes encoding the nitric oxide reductase activation proteins NorD and NorQ, which play a role in denitrification, and carbon starvation protein A (CspA) involved in the carbon starvation stress response.

In view of the above findings, we propose this sequence (CM009897.1) as a chromid, consistent with a previous study [7]. Recently, the presence of chromids has also been reported in other species of the genus *Butyrivibrio*, namely in *B. hungatei* MB2003 [40] and *B. proteoclasticus* B316^T^ [41].

### Conclusion

At present, there are nine genome assembly projects of *B. fibrisolvens* deposited in the NCBI database. Each genome assembled is in more than 60 unordered sequences. The exception is strain 16/4, which is available as a single chromosome, although it appears to be incorrectly classified as a *B. fibrisolvens* strain. An analysis of its 16S rRNA gene sequence and significant differences of its genome metrics when compared with the other *B. fibrisolvens* genomes deposited in the NCBI database support this conclusion.

We consider that INBov1 may serve as a reference genome for *B. fibrisolvens* and propose pINBov266 as a chromid as well. We assembled 96 % of the *B. fibrisolvens* genome in one sequence of 4 398 850 bp and a total of 163 074 unidentified bases, providing the first nearly complete genome sequence and complete genomic structure of *B. fibrisolvens* INBov1. Additionally, we identified and assembled the chromid sequence of 266 542 bp (pINBov266) for this organism by finding the presence of elements – *repA*, *parB* and *hbs* genes *–* characteristic of a plasmidic replication system. These genes are also found co-localized in a region predicted by the GC skew as the probable origin of replication. The designation of pINBov266 as a chromid is also supported by the presence of multiple genes involved in antibiotic resistance and bacteriocin and toxin production. Moreover, the pINBov266 restriction map reveals the absence of any possible alignment between the chromid and chromosome restriction maps. These and other data confirm for the first time the presence of a non-mobilizable chromid in this species. That several genes and functional subsystems are only present in pINBov266 [e.g. aldehyde dehydrogenase (NAD), lyases PL9 and PL11, NiFe hydrogenase maturation, TldE–TldD proteolytic complex, carbon starvation and denitrification genes] suggests that this chromid plays an important and potentially essential role in *B. fibrisolvens*. However, further assays are required to understand the importance of this chromid in the ecology of this bacterium.

As expected, we found that the INBov1 genome encodes a large set of genes involved in the cellulolytic process but does not encode an exoglucanase gene. This is indicative of *B. fibrisolvens* playing an important role in the ruminal fermentation of cellulose as part of the gut microbiome community rather than it being an autonomous cellulolytic microbe. With respect to the hydrogenation of unsaturated fatty acids, no linoleate isomerase gene was found. Nonetheless, the presence of oleate hydratase and enolase genes in the INBov1 genome is consistent with previous studies, indicating that this strain participates in the biohydrogenation of unsaturated fatty acids in the rumen [3]. The oleate hydratase encoded by the INBov1 genome could be part of a resistance mechanism against the bactericidal effect of unsaturated acids, as has been proposed previously [3, 42].

The work described here provides new insight into the genome of *B. fibrisolvens*, contributing to our understanding of a species with high potential in the development of biotechnological applications.

## Data bibliography

1. Rodríguez Hernáez *et al*. Experiment sequencing data. NCBI BioProject PRJNA412083. https://www.ncbi.nlm.nih.gov/bioproject/412083 Sequence Read Archive (SRA) accession SRP128053 (2017).
2. Rodríguez Hernáez, *et al*. Final genome sequences. GenBank Accession Number GCA_003175155.1 (2017).

## Supplementary Data

Supplementary File 1
