# Supplementary material for: The first complete genomic structure of Butyrivibrio fibrisolvens and its chromid
Source: Microb Genom. 2018 Sep 14;4(10):e000216. doi: 10.1099/mgen.0.000216 (PMC6249431; doi:10.1099/mgen.0.000216)
Supplement: Supplementary File 1 [file mgen-4-216-s001.pdf]

Supplementary material - File S1.

Section 1

Bacterial isolation and growth condition

The isolation of *Butyrivibrio fibrisolvens* strain INBov1 was carried out at the Institute of Pathobiology from the National Institute of Agriculture and Livestock Technology (INTA) in Argentina. *B. fibrisolvens* INBov1 was originally isolated from a Holstein cow provided with a ruminal fistula and fed on a lucerne pasture. Rumen contents were homogenized under a CO_2 atmosphere and filtered through two layers of gauze. The strained samples were diluted until 10^{-10} with anaerobic mineral solution (phosphate and mineral salts). For isolation, 0.2 ml of serial dilutions 10^{-7} to 10^{-10} were inoculated into solid pre-reduced media prepared by means of roll-tube technique, based on the roll tubes procedure of Hungate (1966) [1]. These culture media described by Grubb and Dehority (1976) [2] contained 40% clarified rumen fluid, 7% volatile fatty acids (VFA), 0.025% glucose, 0.025% maltose, 0.025% starch, 0.025% cellobiose, 0.25% yeast extract, and 1.5% agar together with other components recommended by Cerón (2014) [3]. The culture was grown in anaerobic conditions, at 39°C during 5 days. Well-isolated colonies obtained from the roll tubes with the highest dilution were subsequently re-isolated. The pure isolated colonies were cultured in liquid medium without ruminal fluids, plus 0.5% glucose, trypticase peptone, yeast extract, calcium carbonate and L-cysteine-HCL and mineral salts (Cerón, 2014) [3]. The culture was grown in anaerobic conditions, at 39°C during 2 days. Bacterial culture in líquid medium was pellet by centrifugation (8000 x 3 min) to perform DNA extraction.

Genome sequencing and assembly

Different de novo assembly strategies were carried out with our complex dataset and then compared, including hybrid assemblies (using Illumina and 454 reads) and single technology assemblies (only Illumina or 454 reads separately). Several assemblers were tested (Newbler, Celera, Velvet, Abyss, SPAdes) and we also compared the usage of pre-trimmed reads to raw reads. Initially, the optical restriction map was used to validate the scaffolds obtained in each assembly trial by using *Soma v2* [4] to evaluate each trial's performance. The results of the alignments of the scaffolds' restriction sites consistent with the optical map (using map

data and *KpnI* restriction sites in scaffolds) were used to calculate the “percentage of map coverage,” which refers to the percentage of sequence assembled which aligns accordingly with the restriction map provided by the optical mapping results.. The percentage of map coverage was used along with the other metrics gathered from the assemblies' output (N50, number and size of contigs and scaffolds, coverage) to determine which trial provided the best result and, consequently, which scaffolds should be used in building the genomic sequence. In a later analysis, we were able to improve the results obtained in *Soma* by carrying out a manual alignment of the unplaced scaffolds using NEBcutter [5] and our own criteria (see File S1 in Supplementary Material) based on the work done by *Valouev et al. (2005)* [6] to decide which unplaced scaffolds to use in the genomic sequence. These criteria uses the error probability in the optical map technique, as explained in Valouev study, to evaluate the alignments of restriction fragments between the unplaced scaffolds and the restriction map.

Genome annotation

The annotation of the genomic sequences was carried out by using the RAST server online [7]. We used the Classic RAST annotation scheme and RAST gene caller. We selected tasks for automatic error correction, frameshifts correction and backfill of gaps.

The genome expected size is estimated by the optical map restriction which resulted in 4,327,514 bp, very similar to the one estimated by the kmer distribution using Illumina PE reads, which is 4,407,001 bp. The optical map expected size is for the main chromosome only, whereas, the kmer spectrum would be for the entire genome content given the genome sequences are present in similar copy numbers. Moreover, using the kmer spectrum distribution, we analyzed how much coverage we obtained with our different sequencing runs; the 454 PE data provided 6X average coverage, and the 454 SE data signified an average coverage of 11X. As for the Illumina PE data, this provided 74X average coverage. All kmer-based estimation was performed as described by the Computational Biology Core of the Institute for Systems Genomics from the University of Connecticut [8].

Phenotypic characterization and 16S rRNA gene analysis

The INBov1 isolate was classified as a *Butyrivibrio fibrisolvens* strain based on its morphologic and metabolic characteristics. We also used EzBioCloud’s database species identification service [9] to confirm the

species identity of INBov1 strain through the analysis of its 16S rRNA sequence.

Growth was achieved only anaerobically. Gram staining showed Gram–negative rod-shaped bacteria and cell motility was observed. INBov1 was able to grow using glucose, cellobiose, maltose, cellulose, pectin, and xylan as carbon source but was unable to utilize starch and cellulose with a strip of filter paper (Whatman N1). Typically, the isolate was able to produce butyric acid. The analysis we conducted using EzBioCloud's database identification service showed that the INBov1 16S rRNA sequence (GenBank: JN642599.1) was most similar to the 16S rRNA gene of the *B. fibrisolvens* strain NCDO 2221^T (ATCC 19171^T), which the EzBioCloud server uses as the *B. fibrisolvens* strain type (GenBank: X89970.1). The sequence similarity value was 98.82% which is higher than 98.7%, the species threshold suggested by several authors for 16S rRNA sequence identity [10] [11]. This result indicates that INBov1 strain is correctly classified as a *B. fibrisolvens* species which is also supported by the genome properties (see "Genome properties and statistics" section in the main article) and the morphologic and metabolic characteristics observed in the culture.

Assembly discussion

We set out to compare the performance of the different assembly methods using our different datasets. We started by assembling all our 454 reads with the Newbler assembler, which is the native assembler of Roche 454 [12], in which we obtained 25 scaffolds, compared to the 28 scaffolds when running Newbler with the Illumina and 454 reads together. Trials with other assemblers, included using the Celera WGS assembler [13] with Illumina reads, followed by scaffolding with 454 mate pair reads using SPAdes [14], which produced in between 200 and 3000 scaffolds with a low map coverage of less than 40% when the scaffolds were aligned to the restriction map (see Table S1 in Section 2). Ultimately, we concluded that the addition of the 454 mate pair reads was vital for scaffolding, and Newbler was by far the best software to scaffold 454 mate pair reads. Moreover, given our dataset, the addition of Illumina reads in a hybrid assembly did not show an improvement in the results using Newbler. In fact, there was a lower performance when running Soma, obtaining a value of ~60% map coverage, as opposed to ~70% when only 454 reads were used. After the manual alignment of unplaced scaffolds, however, both trials presented similar values of map coverage of around 95% (see Table S1)). Even though we were able to obtain a

similar percentage of map coverage with both trials, we kept the assembly of Newbler with 454 data alone in order to leave the Illumina reads for gap-closing in a later analysis. A trial using only 454 SE reads in Newbler produced 226 contigs, demonstrating that the 454 MP reads were vital in producing a much shorter set of 25 scaffolds. Finally, 454 reads were used in raw format since a trial using pre-trimmed reads did not show a better result after further analyses. We used Newbler for the trimming and filtering process because it is the software designed for reads produced by 454 technologies.

Manual alignment of unplaced scaffolds using NEBcutter. Methods and criteria.

We used NEBcutter to generate restriction maps of the unplaced scaffolds with the aim of improving the results obtained in Soma. We uploaded the unplaced scaffolds to NEBcutter website and set the same endonuclease used in the optical map technique (KpnI). In a following step, we mapped the restriction maps of the scaffolds with the optical restriction map of the genome, focusing on those regions where no scaffold was placed by Soma.

The criteria we used took into account the main type of errors present in the optical map technique as described in *Valouev et al. (2005)* [6]. These errors include errors in the calculation of the restriction fragments, missing cuts, missing restriction fragments, false cuts and chimeric reads. Errors in calculation size occur because of uneven distribution of fluorochromes; fragments of more than 4 kb present a standard deviation value of $\delta = 0.55$ (normal distribution model), fragments up to 4 kb follow a different statistical model with values of $\delta = 2.2$. Missing cuts in the map have a frequency of 20% since the efficiency of the endonucleases is 80%. Non-specific activity of the endonucleases causes false cuts in the DNA molecule and statistically follows the Poisson model with a non-specific cut frequency of $\lambda = 0.005$ per kilobase of DNA. Missing restriction fragments in the optical map are very common when fragments are shorter than 2 kb because of the weak adhesion of the fragments to the glass surface during the technique. Another type of error is the one caused by chimeric reads when molecules of DNA of unrelated genomic regions cross. Chimeric reads are hard to identify and they are not addressed in Valouev work. Consequently, a scoring system is usually established to contemplate the chimeric reads in the optical map alignment algorithms.

Taking into account the aforementioned errors, we established the following criteria. We considered that a scaffold's restriction fragment was well aligned with a fragment of the optical map when its size had a

deviation equal to or less than δ = 0.35 for fragments smaller than 4 kb and δ = 2.2 for fragments larger than 4 kb. We used a value of 0.35 instead of the value mentioned in Valouev study (0.55) to obtain results with higher statistic significance. The fragments at both ends of the scaffolds were an exception; they were accepted when their size was inferior to the limit imposed by the standard deviation since the possibility existed that they might be incomplete fragments.

For fragments that did not align, we evaluated whether the misalignment could have been caused by errors in the optical map. When a fragment was missing of a size smaller than 2 kb in the optical map, we considered the scaffold fragment as correctly aligned because of the high frequency of fragments missing smaller than 2 kb. In other misalignments, we evaluated whether the cause could have been missing or false cuts. If the misalignment was from missing or false cuts, we accepted the fragments only if the probability of these errors was greater than 50%. To estimate this probability, two parameters were calculated: “probable number of missing cuts” and “probable number of false cuts.” The first metric was calculated as 20% of the total number of restriction sites present in the map region where the scaffold was being aligned. The second parameter was calculated as the 0.5% of the map region size (in kilobases) where the scaffold was being aligned. When a misaligned fragment could not be caused by any of the technique errors mentioned, we considered that fragment a rejected fragment. We established cut-off values by accepting only scaffolds with more than 90% of fragments and base alignment with the map. Also, more than 90% of the fragments and bases of the map region had to align with the the scaffold. The cut-off values attempted to contemplate other types of errors in the optimal map technique that were hard to identify, such as the problem of chimeric reads and false cuts. According to our criteria, more than 94% of the restriction fragments from each scaffold we accepted aligned well with the map. Also, the restriction fragments that aligned well from a scaffold we accepted always represented more than 98% of the total scaffold bases.

During the process of manual mapping, we found that some large-sized scaffolds aligned accurately with regions of the map where Soma had placed small-sized scaffolds. We considered that the alignment of the large-sized scaffolds was statistically more significant since a larger number of fragments were aligned. As a result, the smaller scaffolds were removed and the larger ones were placed in the genomic sequence instead. The removed scaffolds were later mapped on to the free regions of the map using the same methodology and we were ultimately able to place most of the removed scaffolds in the genome sequence (see following subsection).

5

Results of the manual alignments.

As is shown in Fig.S1, we were able to place six new scaffolds (scaffolds 13, 14, 16, 3, 17 and 6). To place scaffolds 3 and 6, we removed scaffold 20 from region C and scaffolds 18 and 21 from region D. In a following step, we were able to relocate scaffolds 18 and 21 in region C and D, respectively. Scaffold 20 was left out of the final sequence together with the rest of the unplaced scaffolds (8, 19, 21 and 23).

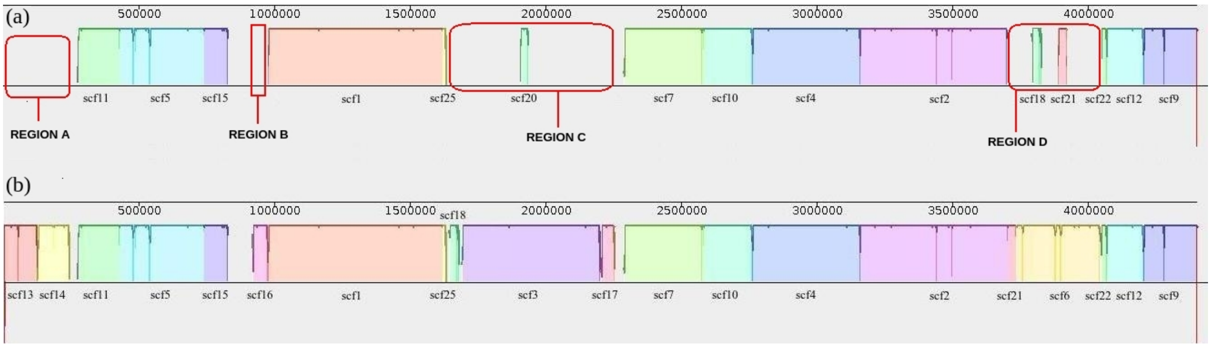

Fig.S1. Progress of the genomic sequence, visualized with Mauve program. Before (a) and after (b) the manual placing of scaffolds with NEBcutter. The regions where new scaffolds were placed are shown (regions A, B, C and D).

The scaffold 8 is a large sequence (266.542 bp) that did not align with any region of the optical map, not even partially. After the annotation process, we also found the presence of repA gene, highly characteristic in plasmids since it codifies an initiator factor that it is essential in the replication system of plasmids [15] [16].

The alignment information of the manually placed scaffolds is shown in the Section 2 of this document.

Construction of the genomic sequence.

After the manual alignment, we calculated the position of the newly placed scaffolds in the optical map as the gap distance present between them and their respective contiguous scaffolds. Using this data, we edited the output table provided by Soma in the alignment to reconstruct the genome sequence. The table indicates the identity and order of the scaffolds placed in the optical restriction map and the gap distance between adjacent scaffolds. An in-house script used the information from

6

the table to place the scaffold sequences and add the gap sequences accordingly. With this script, we created a file with the genome sequence in fasta format. In a final step, we used GapCloser again to close some gaps between the scaffolds using the Illumina PE reads (see Fig.S2).

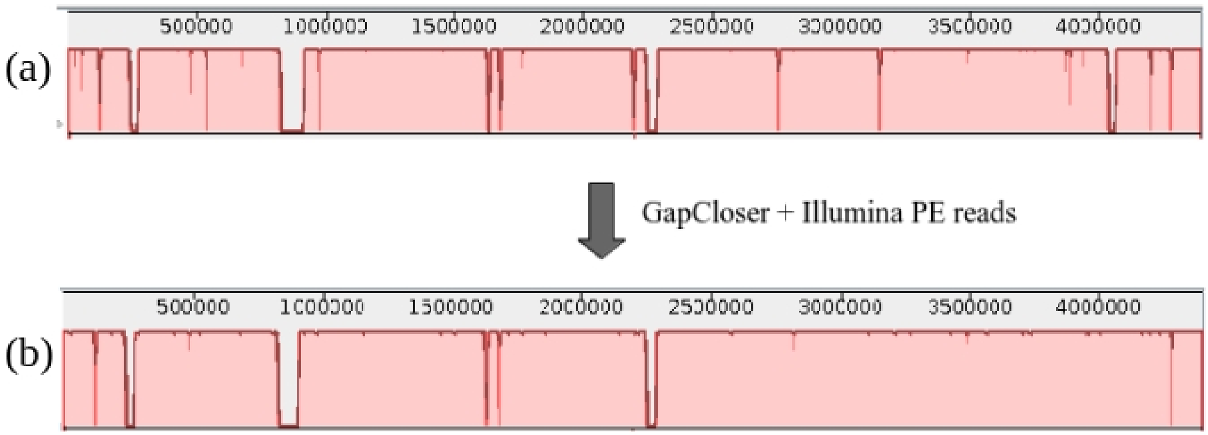

Fig.S2. Progress of the genome sequence before (a) and after (b) closing gaps with the GapCloser program (34% of the gaps were closed). The final genome sequence contains 96% of the estimated genome information. Its size is 4.398.850 bp and it contains 163.074 unidentified bases.

REFERENCES

1. Hungate RE. *The rumen and its microbes. Academic Press, New York and London. 1966.*

2. Grubb JA, Dehority BA. *Variation in colony counts of total viable anaerobic rumen bacteria as influenced by media and cultural methods. Applied and Environmental Microbiology. 1976;31(2):262-267.*

3. Cerón Cucchi M.E. *"Estudio de la diversidad microbiana del compartimento (C1) del sistema digestivo de la llama (Lama glama)". Tesis para optar el título de Doctor de la Facultad de Farmacia y Bioquímica, Universidad de Buenos Aires. Buenos Aires, Argentina. 2014.*

4. Nagarajan N, Read TD, Pop M. *Scaffolding and validation of bacterial genome assemblies using optical restriction maps. Bioinformatics. 2008;24(10):1229-1235. doi:10.1093/bioinformatics/btn102.*

5. Vincze T, Posfai J, Roberts RJ. *NEBcutter: a program to cleave DNA with restriction enzymes. Nucleic Acids Research. 2003;31(13):3688-3691.*

6. Valouev A, Li L, Liu Y, Schwartz D, Yang Y, Zhang Y, Waterman M. *Journal of Computational Biology. April 2006, 13(2): 442-462. https://doi.org/10.1089/cmb.2006.13.442*

7. Aziz RK, Bartels D, Best AA, et al. *The RAST Server: Rapid Annotations using Subsystems Technology. BMC Genomics. 2008;9:75. doi:10.1186/1471-2164-9-75.*

8. Genome Size Estimation Tutorial*. Institute for Systems Genomics from the University of Connecticut < http://bioinformatics.uconn.edu/genome-size-estimation-tutorial > Accesed 2018 January 16.*

9. Yoon S, Ha S, Kwon S, Lim J, Kim Y, Seo H and Chun J. (2017)*. Introducing EzBioCloud: a taxonomically united database of 16S rRNA gene sequences and whole-genome assemblies. Int J Syst Evol Microbiol 67(5):1613-1617 doi:10.1099/ijsem.0.001755*

10. Stackebrandt, E. & Ebers, J. *Taxonomic parameters revisited: tarnished gold standards. Microbiol. Today 8, 6–9 (2006).*

11. Yarza P., Yilmaz P., Pruesse E., Glöckner F. O., Ludwig W., Schleifer K.-H., et al. (2014). *Uniting the classification of cultured and uncultured bacteria and archaea using 16S rRNA gene sequences. Nat. Rev. Microbiol. 12 635–645. doi:10.1038/nrmicro3330*

12. Margulies M, Egholm M, Altman WE, et al*. Genome Sequencing in Open Microfabricated High Density Picoliter Reactors. Nature. 2005;437(7057):376-380. doi:10.1038/nature03959.*

13. Myers EW, Sutton GG, Delcher AL, et al*. A whole-genome assembly of Drosophila. Science. 2000;287:2196–204. doi:10.1126/science.287.5461.2196*

14. Nurk S, Bankevich A, Antipov D, et al. *Assembling Single-Cell Genomes and Mini-Metagenomes From Chimeric MDA Products. Journal of Computational Biology. 2013;20(10):714-737. doi:10.1089/cmb.2013.0084.*

15. Chattoraj DK, Snyder KM, Abeles AL. *P1 plasmid replication: multiple functions of RepA protein at the origin. Proceedings of the National Academy of Sciences of the United States of America. 1985;82(9):2588-2592.*

16. Harrison PW, Lower RP, Kim NK, et al. *Introducing the bacterial 'chromid': not a chromosome, not a plasmid. Trends in Microbiology, Volume 18, Issue 4, 141–148. 2010. doi:10.1016/j.tim.2009.12.010*

 8

Section 2

Table S1. Assembly output metrics.

Input data	assembler	Assembly metrics						SOMA alignments			After manual alignments with NEBCUTTER		
		assembly type	coverage	scaffolds	N50 scfs	contigs	N50 ctgs	seqs placed	bases placed (bp)	map coverage	seqs placed	bases placed (bp)	map coverage
454 SE	Newbler	single technology	11X	-	-	270	39776	46	1989639	45.98%	-	-	-
454 SE trimmed	Newbler	single technology	11X	-	-	277	37617	-	-	< 40,00%	-	-	-
454 SE + 454 MP	Newbler	single technology	17X	25	310360	215	55288	13	2987074	69.03%	17	4157345	96.07%
454 SE trimmed + 454 MP	Newbler	single technology	17X	26	307673	209	55288	14	2984582	68.97%	-	-	-
454 SE + 454 MP + IL PE trimmed	Newbler	hybrid assembly	91X	28	281256	225	79597	14	2518157	58.19%	18	4126807	95.36%
454 SE + 454 MP + IL PE	Newbler	hybrid assembly	91X	28	281130	219	71704	12	1827796	42.24%	-	-	-
454 SE trimmed + 454 MP + IL PE trimmed	Newbler	hybrid assembly	91X	29	304455	231	71750	-	-	< 40,00%	-	-	-
IL PE	Newbler	single technology	74X	130	55331	307	48292	37	2051750	47.41%	-	-	-
IL PE + 454 SE + 454 MP	SPAdes	hybrid assembly	91X	220	-	-	-	-	-	< 40,00%	-	-	-
IL PE	Celera	single technology	91X	582	-	-	-	-	-	< 40,00%	-	-	-
IL PE + 454 SE + 454 MP	Celera	hybrid assembly	91X	1000	-	-	-	-	-	< 40,00%	-	-	-
IL PE + 454 SE + 454 MP	AbySS	hybrid assembly	91X	594	-	-	-	-	-	< 40,00%	-	-	-
IL PE	Velvet	single technology	74X	2946	-	-	-	-	-	< 40,00%	-	-	-

Metrics are not shown when the assembly results showed a poor performance. We considered a poor performance any assembly trial that produced more than 200 scaffolds and showed less than 40% in map coverage after SOMA alignments.
SE: single-end reads **MP:** mate paired reads **trimmed:** reads pre-filtered and processed with Trimmomatic program **IL PE:** Illumina paired-end reads

- Manual alignment information

(a) Manual alignment of scaffolds 13 (upper) and 14 (lower) with region A of the map.

scaffold fragments (kb)	optical map fragments (kb)	standard deviation	diff.	region size (kb)	% deviated	observations	rejected fragments	probable N° of missing cuts	probable N° of false cuts	% accepted fragments	% accepted bases
**SCF13 (Rv)**	**REGION A**										
60.617	42.704	12.811	17.913	107.01	41.95%	Ok (false cut)	0	1	0.54	100.00%	100.00%
6.549	6.718	2.015	-0.169		-2.52%	ok					
37.069	36.704	11.011	0.365		0.99%	ok					
16.269	20.885	6.266	-4.616		-22.10%	ok					
**SCF14 (Rv)**	3.614										
4.013	4.32	1.296	-0.307	119.30	-7.11%	ok	0	2	0.60	100.00%	100.00%
4.827	4.738	1.421	0.089		1.88%	ok					
4.219	4.679	1.404	-0.46		-9.83%	ok					
76.872	78.18	23.454	-1.308		-1.67%	ok					
5.38	7.325	2.198	-1.945		-26.55%	ok					
9.881	10.294	3.088	-0.413		-4.01%	ok					
0.941		-	0.941		-	Ok (missing frag.)					
0.24		-	0.24		-	Ok (missing frag.)					
4.591	1.992<br>7.769	2.928	-5.17		-259.54%	Ok (false cut and end fragment)					

Tables (a), (b), (c) and (d) show, from left to right, the alignment of a scaffold restriction fragments with respective fragments in the map region. Fragment size is measured in kilobases. Rv and Fw provide each scaffold's orientation as it was aligned (**Rv:** reverse; **Fw:** forward). Fragments that aligned well are represented in green color, rejected fragments in red. Missing fragments are the most frequent error found (colored in light blue), the misalignments that were able to be explained because of false cuts or because they were end fragments are shown in orange color. Size deviations higher than 30% in alignments are colored in yellow. The observations column shows the criteria used to evaluate the alignments when such criteria was required.

(b) Manual alignment of scaffold 16 with region B of the optical map.

scaffold fragments (kb)	optical map fragments (kb)	standard deviation	diff.	region size (kb)	% deviated	observations	rejected fragments	probable N° of missing cuts	probable N° of false cuts	% accepted fragments	% accepted bases
**SCF16 (Rv)**	**REGION B**										
0.879	1.715	3.773	-0.836		-48.75%	Ok (Frag. < 4 kb)					
7.629	7.333	2.200	0.296		4.04%	ok					
10.936	10.52	3.156	0.416		3.95%	ok					
0.103		-	0.103	41.63	-	Ok (missing frag.)	0	1.6	0.21	100.00%	100.00%
4.067	4.202	1.261	-0.135		-3.21%	ok					
6.228	6.179	1.854	0.049		0.79%	ok					
11.445	11.683	3.505	-0.238		-2.04%	ok					

(c) Manual alignment of scaffolds 18 (upper), 3 (middle) and 17 (lower) with region C of the map. Scaffold 18 is shown in blue to differentiate it from scaffold 3.

	scaffold fragments (kb)	optical map fragments (kb)	standard deviation	diff.	region size (kb)	% deviated	observations	rejected fragments	probable N° of missing cuts	probable N° of false cuts	% accepted fragments	% accepted bases
	SCF18 (Fw)	REGION C										
	10.095	14.549	4.365	-4.454		-30.61%	ok (end fragment)					
	0.382		0.000	0.382		-	Ok (missing frag.)					
	1.378	2.108	0.632	-0.73		-34.63%	Ok (Frag. < 4 kb)					
	14.226	13.77	4.131	0.456	46.59	3.31%	ok	0	1.4	0.23	100.00%	100.00%
	3.953	4.537	1.361	-0.584		-12.87%	ok					
	3.459	11.629	3.489	-8.17		-70.26%	ok (end fragment)					
	SCF3 (Rv)						gap					
	10.44	10.141	0.203	0.299		2.95%	ok					
	6.319	6.125	0.123	0.194		3.17%	ok					
	17.801	17.371	0.347	0.43		2.48%	ok					
	29.894	29.224	0.584	0.67		2.29%	ok					
	19.784	19.201	0.384	0.583		3.04%	ok					
		2.279	0.046	-0.924		-40.54%	rejected fragment					
	1.355	1.815	0.036	-0.46		-25.34%	ok					
	20.563	19.749	0.395	0.814		4.12%	ok					
	6.482	6.347	0.127	0.135		2.13%	ok					
	16.496	16.504	0.330	-0.008		-0.05%	ok					
	44.66	44.008	0.880	0.652		1.48%	ok					
	4.114	4.224	0.084	-0.11		-2.60%	ok					
	25.981	25.962	0.519	0.019		0.07%	ok					
	3.497	3.739	0.075	-0.242		-6.47%	ok					
	1.198	5.188	0.104	-3.99		-76.91%	Ok (Frag. < 4 kb)					
	13.813	13.377	0.268	0.436		3.26%	ok					
	11.104	10.952	0.219	0.152	418.27	1.39%	ok	2	7	2.09	94.12%	98.81%
	10.304	10.078	0.202	0.226		2.24%	ok					
	20.922	20.022	0.400	0.9		4.50%	ok					
	0.908	1.758	0.035	-0.85		-48.35%	Ok (Frag. < 4 kb)					
	19.298	18.784	0.376	0.514		2.74%	ok					
	13.179	12.819	0.256	0.36		2.81%	ok					
	16.67	16.099	0.322	0.571		3.55%	ok					
	1.767	2.07	0.041	-0.303		-14.64%	ok					
	7.187	7.194	0.144	-0.007		-0.10%	ok					
	5.969	5.919	0.118	0.05		0.84%	ok					
	25.487	24.822	0.496	0.665		2.68%	ok					
	7.329	7.26	0.145	0.069		0.95%	ok					
		2.698	0.054	-2.698		-100.00%	rejected fragment					
	31.907	30.628	0.613	1.279		4.18%	ok					
	6.832	6.774	0.135	0.058		0.86%	ok					
	12.134	12.493	0.250	-0.359		-2.87%	ok					
	0.659		0.000	0.659		-	Ok (missing frag.)					
	2.353	2.643	0.053	-0.29		-10.97%	ok					
	17.64	17.086	0.342	0.554		3.24%	ok					
SCF17 (Fw)	21.574	22.709	0.454	-1.135	92.67	-5.00%	ok	0	0.8	0.46	100.00%	100.00%
	9.464	52.874	1.057	-43.41		-82.10%	ok (end fragment)					

(d) Manual alignment of scaffolds 21 (upper) and 6 (lower) with region D of the map.

scaffold fragments (kb)	optical map fragments (kb)	standard deviation	diff.	region size (kb)	% deviated	observations	rejected fragments	probable N° of missing cuts	probable N° of false cuts	% accepted fragments	% accepted bases
**SCF21 (Fw)**	**REGION D**										
12.011	12.774	0.255	-0.763	29.21	-5.97%	ok	0	1	0.15	100.00%	100.00%
17.24	16.437	0.329	0.803		4.89%	ok					
0.209		0.000	0.209		-	Ok (missing frag.)					
0.41		0.000	0.41		-	Ok (missing frag.)					
**SCF6 (Fw)**											
0.877	3.71	0.074	-2.833	307.43	-76.36%	ok (end fragment)	1	5.4	0.00	96.15%	99.39%
3.783	3.357	0.067	0.426		12.69%	ok					
	1.874	0.037	-1.874		-100.00%	rejected fragment					
4.313	4.535	0.091	-0.222		-4.90%	ok					
0.581		0.000	0.581		-	Ok (missing frag.)					
1.704	1.787	0.036	-0.083		-4.64%	ok					
5.952	5.983	0.120	-0.031		-0.52%	ok					
5.011	4.835	0.097	0.176		3.64%	ok					
21.875	21.318	0.426	0.557		2.61%	ok					
10.249	9.877	0.198	0.372		3.77%	ok					
0.734	1.492	0.030	-0.758		-50.80%	Ok (Frag. < 4 kb)					
18.276	17.75	0.355	0.526		2.96%	ok					
4.493	4.687	0.094	-0.194		-4.14%	ok					
53.999	53.434	1.069	0.565		1.06%	ok					
36.593	37.057	0.741	-0.464		-1.25%	ok					
21.998	21.677	0.434	0.321		1.48%	ok					
0.799	1.957	0.039	-1.158		-59.17%	Ok (Frag. < 4 kb)					
0.013		0.000	0.013		-	Ok (missing frag.)					
44.262	43.207	0.864	1.055		2.44%	ok					
2.93	3.104	0.062	-0.174		-5.61%	ok					
4.525	4.848	0.097	-0.323		-6.66%	ok					
1.085	1.846	0.037	-0.761		-41.22%	Ok (Frag. < 4 kb)					
26.689	25.75	0.515	0.939		3.65%	ok					
12.22	11.742	0.235	0.478		4.07%	ok					
12.884	12.711	0.254	0.173		1.36%	ok					
2.812	2.857	0.057	-0.045		-1.58%	ok					
6.347	6.038	0.121	0.309		5.12%	ok					
